# Nano-Enabled Products: Challenges and Opportunities for Sustainable Agriculture

**DOI:** 10.3390/plants10122727

**Published:** 2021-12-11

**Authors:** Vishnu D. Rajput, Abhishek Singh, Tatiana Minkina, Sapna Rawat, Saglara Mandzhieva, Svetlana Sushkova, Victoria Shuvaeva, Olga Nazarenko, Priyadarshani Rajput, Komariah, Krishan K. Verma, Awani Kumar Singh, Mahesh Rao, Sudhir K. Upadhyay

**Affiliations:** 1 Academy of Biology and Biotechnology, Southern Federal University, 344090 Rostov-on-Don, Russia; tminkina@mail.ru (T.M.); msaglara@mail.ru (S.M.); terra_rossa@mail.ru (S.S.); v_shuvaeva@mail.ru (V.S.); nazarenkoo@mail.ru (O.N.); 2 Department of Agricultural Biotechnology, College of Agriculture, Sardar Vallabhbhai Patel University of Agriculture and Technology, Meerut 250110, India; intmsc.abhi@gmail.com; 3 Department of Botany, University of Delhi, Delhi 110007, India; rawatsapna61@gmail.com; 4 The Smart Materials Research Institute, Southern Federal University, 344090 Rostov-on-Don, Russia; priyadarshanirajput22@gmail.com; 5 Soil Science Department, Faculty of Agriculture, Sebelas Maret University, Surakarta 57126, Indonesia; komariah@staff.uns.ac.id; 6 Guangxi Academy of Agricultural Sciences, Nanning 530007, China; drvermakishan@gmail.com; 7 Centre for Protected Cultivation, ICAR-Indian Agricultural Research Institute, New Delhi 110012, India; singhawani5@gmail.com; 8 Pusa Campus, ICAR-National Institute for Plant Biotechnology (NIPB), New Delhi 110012, India; mraoicar@gmail.com; 9 Department of Environmental Science, V.B.S. Purvanhal University, Jaunpur 222003, India; sku.env.lko@gmail.com

**Keywords:** nanotechnology, nano-insecticide, nano-fertilizer, nano-pesticide, health risk, soil, plant

## Abstract

Nanotechnology has gained popularity in recent years owing to its established potential for application and implementation in various sectors such as medical drugs, medicine, catalysis, energy, material, and plant science. Nanoparticles (NPs) are smaller in size (1–100 nm) with a larger surface area and have many fruitful applications. The extraordinary functions of NPs are utilized in sustainable agriculture due to nano-enabled products, e.g., nano-insecticides, nano-pesticides, and nano-fertilizers. Nanoparticles have lately been suggested as an alternate method for controlling plant pests such as insects, fungi, and weeds. Several NPs exhibit antimicrobial properties considered in food packaging processes; for example, Ag-NPs are commonly used for such purposes. Apart from their antimicrobial properties, NPs such as Si, Ag, Fe, Cu, Al, Zn, ZnO, TiO_2_, CeO_2_, Al_2_O_3_, and carbon nanotubes have also been demonstrated to have negative impacts on plant growth and development. This review examines the field-use of nano-enabled products in sustainable agriculture, future perspectives, and growing environmental concerns. The remarkable information on commercialized nano-enabled products used in the agriculture and allied sectors are also provided.

## 1. Introduction

The world’s population presently stands at almost 7.9 billion (2021), with a significant proportion residing in developing countries, especially in Asia, with approximately 50%. A large bulk of people here confront daily food shortages owing to environmental effects or political uncertainties, whereas in the industrialized world, the drive is to plant drought- and pest-resistant crops, which also boost yields [1,2]. The annual meeting of the American Physical Society was held on 29 December 1959, at the California Institute of Technology, and Richard Feynman delivered a speech entitled “There’s Plenty of Room at the Bottom, An Innovation to Enter a New Field of Physics” [3], which suggested manipulating the matter on an atomic scale and developed the idea of nanotechnology (NT; nano: N, technology: T). However, Norio was the first who used this term in 1974 [4].

Nanotechnology has taken a big step forward, and this is reflected in the money invested in development and research in this field. A budget of $27 billion was proposed by the United States National Nanotechnology Initiative (NNI) in the year 2019; Nano-Mech, a leading NPs manufacturing company, received $10 million from Saudi Aramco Energy Ventures (SAEV); and $350 million was received by the Massachusetts Institute of Technology to develop a state-of-the-art nanoscale center called “MIT nano” [5]. To accomplish the Zero Hunger goal, the United Nations’ aim for sustainable development to be reached by 2030, there is an urgent requirement for thorough variations in conventional agriculture practices. Such changes can be achieved by employing NTs innovations [6,7].

Numerous strategies have been developed by researchers in the last several decades to replace conventional agriculture practices. Of them, NT is one evolving technique that is expected to improve modern agriculture in sustainable ways, such as crop productivity via ameliorating the consequences by increasing plant nutrition, precision farming, water use efficiency, crop protection against pests and diseases by devolving nano-enabled formulations, and environmental restoration of degraded sites by nano-bioremediation [8,9,10]. The distinctive physicochemical properties of NPs greatly help them to be suitable for diverse applications in almost all industries and received extensive attention in agriculture together with numerous other field applications, i.e., biomedical, chemical, optical, pharmaceutical, food, cosmetics, and textile [11]. In addition, NPs are also used in fortifications [7,12] and food processing [13]. 

It is documented that the nano-enabled products are already in commerce and being applied in sustainable agriculture such as nano-fertilizers [14], growth stimulators [15], soil-improving agents [6,16,17], and nano-sensors [18] and have gained popularity as smart and controlled delivery materials. However, the rapid application of NPs has also raised concern for soil microbiota, accumulation in plants, and the threat to human health via the food chain [19]. The review focuses on the possibilities of safe application of nano-enabled products for sustainable agriculture and raises environmental concerns.

## 2. Field Application of Nano-Enabled Products

The practice of applying NPs in agriculture promises a bright future, but it is still in its infancy stage [20]. The synthesis of modified NPs for agricultural purposes has considerable potential to alter the perception of conventional agriculture [21,22]. It helps to solve persisting challenges in agriculture using advanced materials and technologies. Most of these problems are usually related to pesticides, herbicides, fertilizers, etc. and their optimal use. In plants, the distribution of NPs involves a characteristic route with multiple advantages [23].

Nanoparticles have a great capacity to protect plants owing to their essential features such as surface-to-volume ratio, size, and optical attributes [24]. A nice example of NPs application is nano-encapsulated fertilizers and pesticides dispensed via a method that distributes the content in a controlled manner [25,26]. This method is also site-specific. For the protection of non-target organisms and other collateral repairs, this highly efficient method is beneficial [27] and provides an effective defense mechanism when plants engage with pathogens by sending systemic compounds such as jasmonic acid, salicylic acid, benzothiadiazole, and others to the appropriate sites [28,29]. 

**Figure 1:**
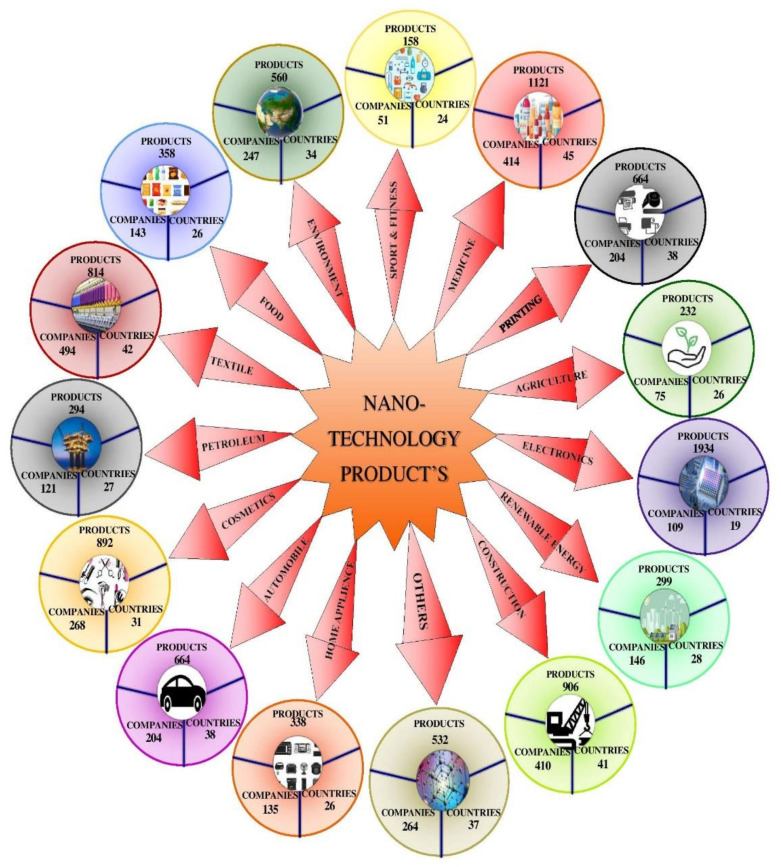
Representation of nanotechnology products, manufacturing firms, and countries involved.

In agriculture, some other applications of NT can be seen, such as attenuating natural pesticides and biopesticides, and the measured and controlled release of fertilizers supported by NPs, micronutrients, nano-sensors, and organic fertilizers [14,18,30]. The nanotechnology products database (NPD) website, used to keep updated records on nano-enabled products, showed the total number of products to be 9245 manufactured by 64 countries via 2658 companies (https://product.statnano.com/; accessed on 7 July 2021) in commercial production. Figure 1 illustrates various nano-enabled products and their application in different fields such as agriculture, automotive, construction, cosmetics, printing, petroleum, environment, textile, electronics, sports, fitness, environment, home appliances, renewable energy, food, medicine, and other nano-enabled products. 

In agriculture, more than 232 nano-enabled products are manufactured by 75 companies in 26 countries all over the world. These nano-enabled products are related to plant protection, fertilizers, soil improvement, plant breeding, and animal husbandry. Figure 1 illustrates applications of nano-enabled products in different fields, including agriculture. Similarly, in automotive industry manufacturing, a total of 664 nano-enabled products are manufactured by 204 companies in 38 countries. These nano-enabled products used in automotive industries are related to auto-additives, auto-parts, and maintenance. In the same way, more than 906 nano-enabled products produced by 410 companies in 41 countries are in use in construction industries. In the cosmetic industry, a total of 892 nano-enabled products are in use; in printing, 664; in textiles, 814; in electronics, 894; and in pharmaceutical companies, 1121 nano-enabled products are in use (Figure 1). Thus, nano-enabled products have a wide range of applications in various industries as described in Figure 1.

### 2.1. Nano-Enabled Products and Crop Protection

Nowadays, fungicides, pesticides, herbicides, and fertilizers are applied in soil in inappropriate amounts through foliar application. Fungicides, pesticides, and herbicides are a mixture of chemical substrates that can be used to control, prevent, or destroy any pests, microbes, or unwanted herbs that infect crops and reduce crop production [22,31,32]. Algicides, herbicides, fungicides, pesticides, and plant disinfecting agents are the main nano-enabled products considered for crop protection nowadays. More recently, nano-based stretch films made with polyethylene have been developed for hay packing to protect crops against UV-rays and thunder and have been used in active food packaging systems to protect against *Escherichia coli* spoilage [33]. The 40 plant-protection, nano-enabled products are available in the commercial market in 12 countries and were developed by 16 companies. Among all 40 products, 13 products are widely used for crop protection and disease management (Table 1). 

#### 2.1.1. Nano-Pesticides

Pesticides kill unwanted, undesirable substances like fungi, algae, and bacteria, and make plants resistant to them. The major difficulty associated with pesticides is their solubility because they are not easily soluble in water and release very slowly after spraying, thus making them ineffective [34]. Therefore, with the aid of NT, nano-pesticides enclosed within a shell made with the support of NPs are being prepared [35,36]. These nano-pesticides reduce soil run-off while also increasing solubility [35,37].

#### 2.1.2. Nano-Insecticides

Some insecticides are not fully soluble in water and thus, must be dissolved in organic solvents, making them toxic and also increasing the cost of the pesticide [34,38,39]. Nevertheless, the use of NPs could enhance the solubility of insecticides besides reducing toxicity. Modified chitosan and porous silica-based NPs have been effectively loaded into insecticides, and less water-soluble pesticides can be prepared [35,37,40]. In *Spodoptera litura* ovarian cell lines, hydrophobic insecticides such as azadirachtin with improved chitosan NPs demonstrated a promising decrease in cell proliferation and the sustained release of drugs [41]. The potential advantages of NPs were underlined in all of this research. The use of a solid–lipid mixture of NPs also solved the evaporation, or volatilization, issue correlated with the post-application of insecticide [42,43]. Insecticides applied as a mixture with NPs were observed to be useful in slowing down the release of active molecules that could help to reduce the toxic impacts of insecticides [44,45]. 

#### 2.1.3. Nano-Fungicides

The most explored and beneficial NPs as fungicides are chitosan, silica, and polymer mixtures [31,46]. The efficacies of nano-fungicides have been noted against a variety of fungal species; however, they have rarely been tested on the crops without any toxic effects [47]. Generic insecticides possess problems such as low water solubility, poor stability, and a volatile nature that may disturb the sustained release [42]. These issues could be resolved by applying NPs-based fungicides. Low-water-soluble fungicides such as chitosan-lactide copolymer were loaded onto NPs in various concentrations, and their effectiveness was monitored as a growth inhibitor of *Colletotrichum gossypii* [48]. An eco-friendly alternative to current synthetic fungicides is predicted to play a major role in future plant disease management [31,42,49]. 

In vitro tests for some repressive features of NPs such as Cu-NPs, CuO-NPs, Ag-NPs, and ZnO-NPs against economically relevant foliar and soil-borne plant diseases were conducted [42]. The Cu-NPs were found to be the most effective at inhibiting mycelial development (EC50 values of 162 to 310 μg/mL), followed by ZnO-NPs (EC50 values of 235 to 848 μg/mL), and were practically insensitive to all fungal species, whereas Ag-NPs had a considerable inhibitory effect on *Botrytis cinerea* [50]. Furthermore, when NPs and their bulk equivalents were evaluated for antifungal activity, ZnO-NPs were found to be more effective against all fungal species than ZnSO_4_. The Cu-NPs were also shown to be more effective than CuSO_4_ against all species, excluding *B. cinerea*, *A. alernata*, and *M. fructicola* [50]. The absence of any link between NPs and their corresponding bulk metal and the positive association between Cu-NPs and CuO-NPs toxicity indicates potential changes in the mode of action between bulk- and nano-sized metals [42]. Although there is considerable diversity between fungal species, the toxicity of all NPs rose significantly when applying to spores instead of fungal hyphae [51]. This reveals that these compounds have a high potential for application as protective anti-fungal treatments. 

#### 2.1.4. Nano-Herbicides

Herbicides are used to kill weeds and unwanted grasses. They possess high levels of toxicity and a long half-life and are frequently unaffected by standard treatment. Generally, weeds grown along with crops affect the growth and yield of crops, and hence, traditional herbicides are applied to restrict the growth of undesired weeds. However, the application of herbicides might affect plant growth and development [52]. In this context, nano-herbicides can be effectively used to solve such type of issues [53,54]. The nano-herbicides are easily dissolved in soil particles and kill the weeds and could be designed in a form that is less hard on crops. 

Nano-enabled herbicides are shown to have the remarkable potential to destroy weeds and improve crop yields [53,55]. Moreover, NPs applied as a mixture with certain herbicides such as atrazine, ametrine, triazine, and the effectiveness of these nano-enabled herbicides improved by 84% [56,57]. A commonly used herbicide to eliminate weeds and unwanted grasses is atrazine [58]. Once weeds are destroyed by herbicides, the nutrients available in the soil remain solely accessible for plants; consequently, plants develop resistance to diseases caused by nutrient deficiencies [55]. Moreover, the use of modified NPs in combination with carboxymethyl cellulose could also improve pesticide breakdown efficiency [59,60]. 

#### 2.1.5. Nano-Fertilizer

Nutrient use efficiency (NUE) is a worldwide concern due to the poor efficiency of applied nutrients [61]. Fertilizers are vital agricultural products that enhance crop production. In farming ecosystems, fertilizer contributes up to 35–40% of the productivity of any crop [62]. Without the application of fertilizers in crops, it becomes difficult to expect the required production. For this reason, a large amount of fertilizer is applied to crops in every country [63]. Due to the subsidy on fertilizers, the excess use of fertilizer has increased the amount of nitrates in groundwater, which causes nitrate pollution. In the last few years, use efficiencies of N, P, and K fertilizers remained constant as 30–35%, 18–20%, and 35–40%, respectively, leaving a higher portion of added fertilizers to stay in the soil or enter the aquatic system causing eutrophication [25,64].

To resolve the problem of fertilizer accumulation, nano-enabled fertilizers could be used by regulating the release of nutrients depending on the requirement of crops. However, now, few nano-fertilizer formulations are being synthesized in China, Germany, and the USA, and few are being tested under laboratory conditions [47]. The use of nano-fertilizers can increase the NUE and prevent environmental hazards [65]. Recent works also focused on carbon nanotube-based fertilizers [66]. Carbon nanotubes carry extensive surface area and could regulate moisture under the constraints of irrigation or drought conditions [47,67]. At present, more than 102 nano-enabled fertilizer products are available in the commercial market in 17 countries and have been developed by 41 companies. Among all 102 products, five nano-products are widely used in crop production (Table 2). 

## 3. Soil Health Improvement

These days people know that NPs are in the soil system [17,62]. After a very thorough study of the soil, researchers have found a new class of clay particles, called “Nanosols”—those ranging in size from 1–100 nm—that are more diverse than the other soil clay particles [62]. De-novo synthesized NPs have novel properties and characteristics, i.e., a very high specific surface area (SSAs) and tiny, subsequent surface charges resulting in these NPs reacting very actively with other particles in the soil matrix [62]. Due to their unique properties, NPs have a higher resistance nature, do not decompose rapidly, and accumulate slowly in the soil [17,68]. The aggregative nature of NPs could affect the properties of the soil. To decrease NPs’ toxic effects, it is better to combine NPs with clay as a colloidal solution [69]. The constant use of urea, herbicides, and pesticides in the soil damages both the fertilizer capacity and soil health [70,71]. As the amount of urea, herbicides, and pesticides in the soil gradually rises above a certain concentration, they act like toxic pollutants and become harmful for both the environment and humans. 

Recent studies indicated that the application of NPs improved soil enzymatic activities. Up to 200 mg kg^−1^ carbon NPs significantly improved the activities of urease, dehydrogenase, and phosphatase [72], and it also improved the storage capacity of soils for available nutrients such as ammonium N, nitrate N, and available P [72]. Multi-walled carbon nanotubes showed a stimulative impact on these soil enzymes (urease, phosphatase, and dehydrogenase) [73]. NPs can reduce the danger of soil and play a decisive role in soil management. For soil management, more than nano-enabled products are normally available on the commercial market and can be found in three countries as developed by three companies. These companies’ products (Descal, Humic 101, Coco: flower, Coco: grow, Hydro: flower, Soilklik) do not add sufficient details regarding product applications.

## 4. Plant Uptake and Translocation of Nano-Enabled Products

**Figure 2:**
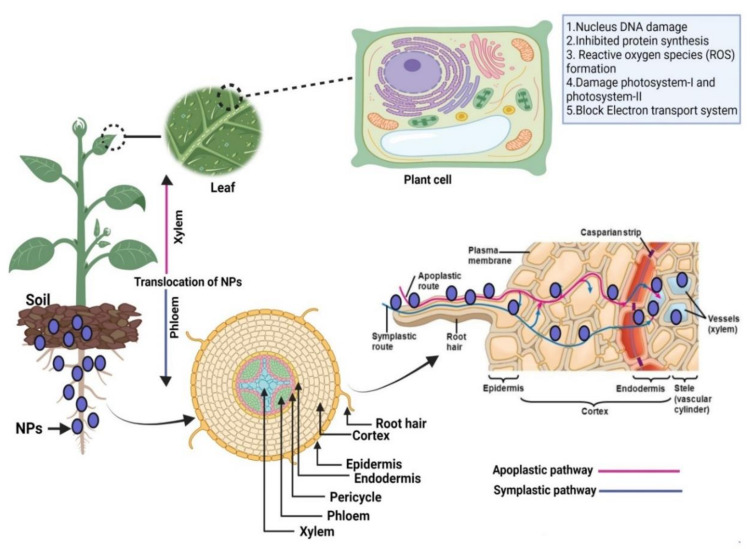
Uptake and translocation of nanoparticles by plant tissues: nanoparticles interact with plant roots, translocate through the epidermis, cortex, endodermis, and pericycle and then into xylem vessels via apoplastic and symplastic pathways.

This section discusses plants’ uptake of water and dissolved solutes from the root to the top of the plant through translocation. The transpiration–cohesion–tension mechanism and root pressure play a major role in raising water to the top of plants through the vascular tissue system, primarily from xylem. Soil solutions with higher water potential compared to root hair cells pass into cortical cells, through the endodermis and the pericycle, and then into the xylem vessels. This whole process of translocation depends on water potential. Likewise, the exposure of NPs in the soil first interacted with the plant cell wall and cell membrane of the root epidermis. Through a complex plant uptake mechanism, NPs enter into the plant vascular bundle (xylem) and translocate into leaves (Figure 2). The absorption of NPs from the soil is also considered a primary step of the bioaccumulation of NPs into the plant system [74]. Once NPs penetrate the plant cells and are transported into the plant tissue, there are two pathways for transportation: the apoplast and the symplast [75].

In the apoplastic pathway, NPs are transported outside the plasma membrane through the extracellular spaces, cell walls of adjacent cells, and xylem vessels [76]. Symplast transportation movement of NPs takes place between the cytoplasm of adjacent cells through specialized structures called plasmodesmata and sieve plates [77]. 

The translocation of NPs through apoplastic pathways is essential for radial movement within plant tissue that allows NPs to reach the vascular tissue system that aids in further movement of NPs through the xylem in various parts of the plant tissue [75,78,79]. As the application of NPs is increasing in agriculture, it has become very important to know how they are transported and interact with plants’ roots and their organelles. Accurate information regarding the translocation pathways of NPs in plant cells could provide an improved understanding of the transportation of NPs via phloem or xylem. It could also help to understand their accumulation in plant organs acting as sinks, such as fruits and grains, so that in the future, it is easy to detect NP accumulation and assess the toxicity and to avoid it by designing nano-enabled products to reduce the accumulation of NPs in plant cells. Consequently, it could be used to reduce their effects, such as altering various biological processes (Figure 2). 

## 5. Conclusions and Concerns Associated with Nano-Enabled Products

The major concerns that arise with nano-enabled products are about the potential concentration of NPs used because, at higher concentrations, NPs showed toxic effects at various levels. The continuous application of nano-enabled products, especially in agriculture, could increase their content in soil and in crops. Most of the nano-enabled products shown in the tables/figure do not reveale the used concentration, particle size, name, and synthesis methods of applied NPs. Nanoparticles could be transferred from root to leaf and leaf to root, thus entering into the food chain. Moreover, they exhibit large-scale bioretention and accumulation within living organisms, possibly beyond safe levels. Even a certain quantity of NPs could be harmful to human health. Higher levels of toxicity can cause growth retardation and inhibition. However, the toxicity of NPs is specific to the shape, size, concentrations, base materials, and coatings. There is an extensive range of nano-enabled products in use that could be broken down into a different class of compound such as metals, metal-oxides, carbon, and semi-conductor materials and could generate new types of hazardous pollutants. To address these issues, green nanotechnology is a multidisciplinary field that is creating clean, safe, and environmentally friendly alternatives to products being currently used in various different industries. As their name signifies, they are derived from different plant parts, microorganisms, amino acids, glucose, etc. They suggest improving the commercial readiness of these technologies. The current review explored the use of nano-enabled products and discovered some interesting features based on recent findings. However, additional scientific evidence regarding these or new nano-enabled products is required. The studies should be directed towards determining the underlying mechanisms of nano-enabled products’ interplay with the food chain, as well as their epigenetic consequences.

## Figures and Tables

**Table 1:** Number of nano-enabled products, manufacturing firms, and the countries involved in plant-protection related products.

Nano-Enabled Products, Manufacturing Firm	* NPs	Properties	Applications
Nanocu; BioNano Technology, Egypt	Cu	Anti-fungal activity properties	Use as an antifungal agent with growth enhancer and improve root vigor property
Nano Cube^®^ Marinus Complete Plus 30l; Dennerle, Germany	Ag	Algicidal properties	Applicable for management of algae
Blue Lagoon UV-Block; Dennerle, Germany	Ag	Algicidal properties	To prevent algal infection and growth in agriculture ponds
Nanosept^®^ Aqua Super; Nanosept, Hungary	Ag	Bactericide, fungicide and virucide	Broad-spectrum plant protectant, control of yield-robbing diseases
Nanosept^®^ Aqua; Nanosept, Hungary	Ag	Disinfecting agent	Broad-spectrum antimicrobial applications

* Note: detailed information on used NPs is missing such as concentration and particle size of NPs.

**Table 2:** Number of nanoproducts, companies, and the countries that are involved in the nano-fertilizer related nano products development.

Nano-Enabled Products, Manufacturing Firm	* NPs	Properties	Applications
Nano Fertilizer; Silvertech Kimya Sanayi ve Ticaret Ltd., Turkey	ZnO, TiO_2_	Plant growth acceleration, Crop yield enhancement, Photosynthesis improvement	Increase the use efficiency of plant nutrients and reduce the soil toxicity
Nubiotek^®^ Hyper Fe+Mg; Bioteksa, Mexico	Fe, Mg	Nubiotek^®^ Hyper Fe+Mg nano-enabled fertilizer functions as activator improve the vegetative development	It ensures sustained growth of plants in all the phenological stages, without negative incidences in the production due to environmental factors
Nubiotek^®^ Ultra Ca; Bioteksa, Mexico	Ca	Nubiotek^®^ Ultra Ca effectively helps the crop, in general, tolerate different scenarios of climatic stress, aggressive presence of pests or diseases with great performance	An enantiomorphic, amiphyloid and coloid that allows the Ca element to penetrate quickly and effectively into the tissues of the crops
Fértil Calmag; HPL Agronegocios, Brazil	Mg	Product with a high concentration of Mg with high-tech liquid solution of NPs	Mg quelatizados allow a greater solubility without decantation, has a high concentration and high availability of nutrients for plants with immediate reaction
Nanovec TSS 80; Laboratórios Bio-Médicin, Brazil	Mg, Mo, Zn	Nanovec TSS 80 nanocapsules composed of Mg, Mo, and Zn compatible with plant cells and transport nutrients into the plants without loss	Useful for leguminous plants, improve germination, root systems and absorbent radicels; the plant absorbs more water and nutrients from the soil, resisting drought periods; increase number of nodules. It transforms the nitrate into protein, improving the use of N and biological fixation of nitrogen
Fertile Calcium 25; HPL Agronegocios, Brazil	Ca	Fértil Calcium 25 is easy to apply, it neutralizes the phytotoxic effect of Al	It is a fluid fertilizer with a high concentration of Ca with rapid root absorption; it prevents diseases in Ca deficient areas
Lithocal, Litho Plant, Brazil	Ca, Mg	It is composed of nano-Ca and Mg particles, recommended for application directly to the soil, in the form of spraying in total area	An essential elements for crops, promoting root system, enhance nutrients uptake, better resisting the periods of drought, increasing tillering sprouts and vegetative vigor
Fosvit K30; Kimitec Group, Spain	P, K	Fertilizer with a high content of P (30%) and K (20%)	A self-defense enhancer against fungal diseases such as omicosis (mildew), promotes the formation of phytoalexins, it distribution K within plant tissues
Nano Bor 20%; Alert Biotech, India	B	Used as micronutrient	It is useful for extreme rainfall regions with acidic and sandy soil with deficient of organic matter
Nano Zinc (Soil Application 21%); Alert Biotech, India	Zn	This product is intended for dealing Zinc deficiency related issue of crops	It prevented the chlorosis of rice and was very effective in curing all such deficiency-related issues
Nano Nutrients for Crops; NanoLandBaltic, Lithuania	SiO_2_	Product contains particles 3–50 nm	Used for crops, flowers, trees, and creepers, when it is sprayed on to leaves it delivers all nutrients to chlorophyll directly and enhance the efficiency of photosynthesis
Nano Zinc Chelate Fertilizer; AFME TRADING GROUP, United Kingdom	Zn	Growth enhancer	It provide one or more nutrient metal elements to plants and increase the growth and development
Nano Iron and Calcium, Potassium Chelate Fertilizer; Afme Trading Group, United Kingdom	Fe, Ca	Plant growth regulater and accelerater	Helps to make up for Fe/Ca/K deficiency
Nano-Ag Answer^®^; Urth Agriculture, USA	Ag	Useful for plant and soil microorganism	It is help in plants and mycorhizal fungi to uptake the nutrients and eventually contribute more organic matter fixation for plants

* Note: detailed information on used NPs are missing such as concentration and particle size of NPs.

## Data Availability

Not applicable.
